# β-Carbonic Anhydrases Play a Role in Fruiting Body Development and Ascospore Germination in the Filamentous Fungus *Sordaria macrospora*

**DOI:** 10.1371/journal.pone.0005177

**Published:** 2009-04-13

**Authors:** Skander Elleuche, Stefanie Pöggeler

**Affiliations:** 1 Institute of Microbiology and Genetics, Department of Genetics of Eukaryotic Microorganisms, Georg-August University, Göttingen, Germany; 2 Faculty of Biology and Biotechnology, Ruhr-University of Bochum, Bochum, Germany; Cinvestav, Mexico

## Abstract

Carbon dioxide (CO_2_) is among the most important gases for all organisms. Its reversible interconversion to bicarbonate (HCO_3_^−^) reaches equilibrium spontaneously, but slowly, and can be accelerated by a ubiquitous group of enzymes called carbonic anhydrases (CAs). These enzymes are grouped by their distinct structural features into α-, β-, γ-, δ- and ζ-classes. While physiological functions of mammalian, prokaryotic, plant and algal CAs have been extensively studied over the past years, the role of β-CAs in yeasts and the human pathogen *Cryptococcus neoformans* has been elucidated only recently, and the function of CAs in multicellular filamentous ascomycetes is mostly unknown. To assess the role of CAs in the development of filamentous ascomycetes, the function of three genes, *cas1*, *cas2* and *cas3* (carbonic anhydrase of *Sordaria*) encoding β-class carbonic anhydrases was characterized in the filamentous ascomycetous fungus *Sordaria macrospora*. Fluorescence microscopy was used to determine the localization of GFP- and DsRED-tagged CAs. While CAS1 and CAS3 are cytoplasmic enzymes, CAS2 is localized to the mitochondria. To assess the function of the three isoenzymes, we generated knock-out strains for all three *cas* genes (Δcas1, Δcas2, and Δcas3) as well as all combinations of double mutants. No effect on vegetative growth, fruiting-body and ascospore development was seen in the single mutant strains lacking *cas1* or *cas3*, while single mutant Δcas2 was affected in vegetative growth, fruiting-body development and ascospore germination, and the double mutant strain Δcas1/2 was completely sterile. Defects caused by the lack of *cas2* could be partially complemented by elevated CO_2_ levels or overexpression of *cas1*, *cas3*, or a non-mitochondrial *cas2* variant. The results suggest that CAs are required for sexual reproduction in filamentous ascomycetes and that the multiplicity of isoforms results in redundancy of specific and non-specific functions.

## Introduction

Carbon dioxide (CO_2_) and its hydration product bicarbonate (HCO_3_^−^) are essential molecules in various physiological processes. In all domains of life, from microorganisms to mammals, CO_2_ is the end-product of respiration. Plants, algae and cyanobacteria are able to fix CO_2_ during photosynthesis. Although it represents only 0.036% of the atmospheric gases, CO_2_ is found at concentrations of roughly 5% in the human bloodstream and in tissues, where respiration takes place. CO_2_ is able to diffuse through lipid-containing cell membranes, but bicarbonate is negatively charged and not permeable to lipid bi-layers [1], [2]. Since the reversible interconversion of CO_2_ and HCO_3_^−^ is spontaneously balanced, CO_2_ is in equilibrium with HCO_3_^−^ and carbonic acid. The reaction CO_2_+H_2_O ↔ HCO_3_^−^+H^+^ is slow, but can be accelerated by the ubiquitous enzyme carbonic anhydrase (CA; [EC 4.2.1.1]). CAs are typically Zn^2+^-metalloenzymes. Based on their amino acid sequence and structure they can be divided into five distinct classes, α, β, γ, δ and ζ, that share no sequence similarity, and appear to have evolved independently [3]–[5]. At least 16 different α-CA isoforms have been isolated in mammals, while plants and fungi encode α- and β-class carbonic anhydrases [3], [6], [7]. The γ-class is predominantly distributed in archaea and eubacteria, but has also been identified in the mitochondria of plants [8], [9]. The ζ- class, which has a cadmium center and the δ-class have been discovered in marine diatoms [10], [11].

To date, only ß-CAs have been characterized in fungi. The function of a β-CA in the hemiascomycetous yeasts *Saccharomyces cerevisiae* and in the human pathogen *Candida albicans* has been elucidated. In both, deletion of the β-CA-encoding *NCE103* gene resulted in a mutant that required elevated CO_2_ levels for growth [12]–[14]. The *S. cerevisiae* mutant could be functionally complemented with a plant β-CA from *Medicago sativa* [14]. In addition, expression of the *S. cerevisiae NCE103* gene was dramatically up-regulated under ambient air conditions [12]. In *C. albicans* the β-CA functions as a CO_2_ scavenger essential for pathogenicity in niches where the available CO_2_ is limited [13]. In contrast to *S. cerevisiae* and *C. albicans*, the basidiomycetous human pathogen *Cryptococcus neoformans* encodes two β-CAs whose functions have recently been elucidated [15], [16]. Similar to *C. albicans*, growth and morphogenesis of *C. neoformans* is strongly influenced by CO_2_, which is directly sensed by the bicarbonate-regulated adenylyl cyclases [13], [15]. The *CAN2* gene of *C. neoformans* was shown to encode its major CA. Its activity is essential to survival and proliferation as well as for basidia and basidiospore formation, but is dispensable for lethality during infection [16].

Similar to *C. neoformans*, filamentous ascomycetes seem to contain multiple β-CAs: three β-CA homologues have been identified in the genome of the ascomycete *Aspergillus fumigatus* [7]. However, the functional role of β-CAs in filamentous fungi has not yet been assessed and it is still not known if the multiplicity of isoforms is related to organelles or cell- or tissue- specific expression and function during development. Hence, we were interested in assessing the functional role of CAs in filamentous ascomycetes.

Here, we present a genetic analysis of three β-CAs from the model organism *Sordaria macrospora*, which has been intensively used to study sexual reproduction and fruiting-body (perithecium) differentiation [17]–[22]. *S*. *macrospora* is a coprophilous fungus that lives on the dung of herbivores. It is a close relative of *Neurospora crassa*, but is in contrast to the heterothallic (self-sterile) *N. crassa*, homothallic (self-fertile) [23], [24]. Under laboratory conditions, *S. macrospora* completes its life cycle within seven days and produces pear-shaped fruiting-bodies with asci and ascospores. A genetic advantage of *S. macrospora* is that recessive mutations can directly be tested for impairment in fruiting-body development, without the need of crossing strains of opposite mating-types [25].

In this study we identified three genes encoding β-CAs (*cas1*, *cas2* and *cas3*) in *S. macrospora*. Localization studies revealed that CAS2 was translocated into the mitochondria, while CAS1 and CAS3 are cytoplasmic. A genetic analysis of knock-out strains, Δcas1, Δcas2, and Δcas3, demonstrate that CAS2 may be the major CA in *S. macrospora*, involved in vegetative growth and ascospore germination.

Developmental defects were severely increased in a Δcas1/2 double deletion strain, which was completely sterile and produced a reduced number of empty fruiting-bodies only after 21 days of growth. The defect in fruiting-body development, but not the decrease in germination efficiency, could be complemented by elevated CO_2_ (5%) concentrations. Interestingly, we were also able to complement the delay in fruiting-body formation in the Δcas1/2 strain by overexpressing either *cas1*, *cas3*, or a cytoplasmically-located *cas2* variant, indicating that the function of CAs is redundant for fruiting-body formation in *S. macrospora*.

## Results

### Identification of three carbonic anhydrase genes in *S. macrospora*

**Figure 1:**
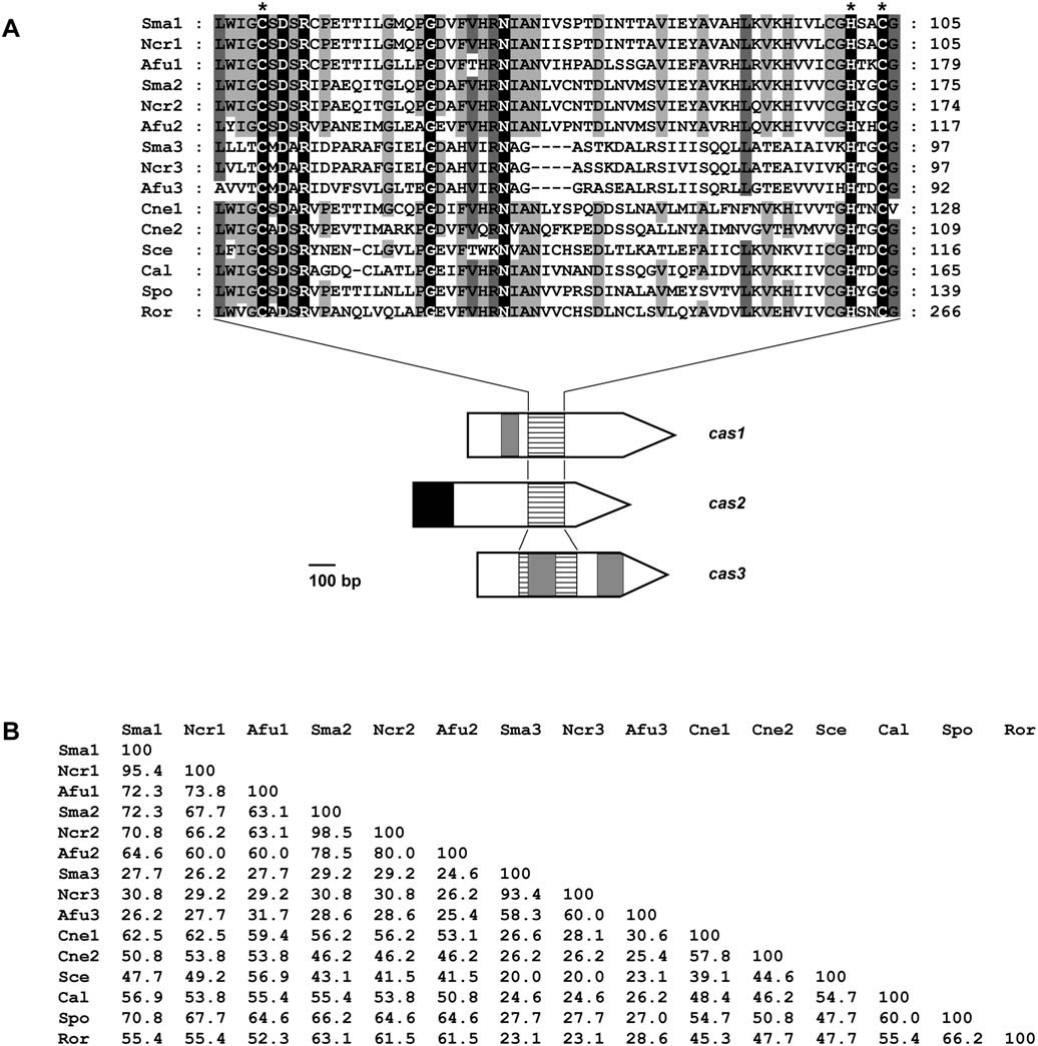
Multiple sequence alignment of the zinc coordinating region from fungal β-class CAs. (A) ClustalX alignment was created using the following sequences: Sma1 [*S. macrospora*, Accession No. FM878639], Sma2 [FM878640], Sma3 [FM878641], Ncr1 [*Neurospora crassa*, Q7S631], Ncr2 [Q7S4J8], Ncr3 [Q8X0H0], Afu1 [*Aspergillus fumigatus*, Q4WQ18], Afu2 [A4DA32], Afu3 [Q4WPJ0], Cne1 [*Cryptococcus neoformans*, Q3I4V7], Cne2 [Q30E79], Sce [*Saccharomyces cerevisiae*, P53615], Spo [*Schizosaccharomyces pombe*, O94255] and Ror [*Rhizopus oryzae*, RO3G_10751.1]. Conserved amino acids important for Zn^2+^-coordination are marked by an asterisk. Identical amino acids, which are conserved in all proteins, are shaded in black; residues conserved in at least 13 of 15 sequences are shaded in dark grey and residues conserved in at least ten sequences are shaded in light grey. Arrows indicate *S. macrospora cas* genes with introns are given as grey boxes. The dashed box marks the region encoding the part of the protein which was used for the alignment at the top. The coding region for the mitochondrial target sequence of CAS2 is indicated as black box. (B) Amino acid identity in % is given for all sequences in pair-wise comparisons. Percentages given are based on amino acid comparison of the conserved region shown in (A).

*S. macrospora* and *N. crassa* are closely related and share a high degree of nucleic acid identity within open reading frames. Therefore, the *N. crassa* database is often used to identify homologous genes in *S. macrospora*, whose genome is not yet sequenced [24]. Database searches with the *S. cerevisiae* CA NCE103p revealed the presence of two putative CA genes in the genome of *N. crassa.* The enzymes are encoded by the ORFs *NCU04778.3* and *NCU08133.3*. A BLASTP search using the catalytic region of the putative CA NCU08133.3 resulted in the identification of a third CA encoded by *NCU01103.3*. Using heterologous primers designed from the *N. crassa* CA ORFs, we succeeded in isolating three putative CA genes from *S. macrospora*. Sequence alignments with several fungal CAs assigned the three *S. macrospora* CAs to the β-class. Three highly conserved residues function as zinc-binding ligands (Figure 1A). Based on these homologies, the three *S. macrospora* genes were termed *cas1* (carbonic anhydrase of *Sordaria macrospora*; *NCU04778.3* homologue, accession number FM878639), *cas2* (*NCU08133.3* homologue, accession number FM878640) and *cas3* (*NCU01103.3* homologue, accession number FM878641). The conserved β-CA domain is located in the central part of CAS1 and CAS3, and in the C terminus of CAS2 (Figure 1A). In *cas3,* the coding region of the conserved domain is interrupted by an intron. To confirm the splicing of predicted introns, cDNAs of the three *cas* genes were generated by RT-PCR with primer pairs cynT1-pQE-f/cynT1-r; cynT2-pQE-f/cynT2-r and cynT3-pQE-f/cynT3-r, respectively. We confirmed the presence of one predicted intron within the 770 bp coding region of the *cas1* gene (65 bp), and two predicted introns within the 733 bp coding region of the *cas3* gene (103 and 105 bp). Sequence analysis of the 855 bp *cas2* coding region revealed that this gene is not interrupted by an intron. The deduced peptide sequences of the putative *S. macrospora cas1*, *cas2* and *cas3* cDNAs encode for 234, 284 and 174 aa proteins with predicted molecular masses of 25.1, 32.1 and 19.1 kDa, respectively, and theoretical isoelectric points of 6.16, 8.15 and 5.16. cDNAs of the three *S. macrospora cas* genes fused to either RGS-His-tag or GST-tag were heterologously expressed in *E. coli*. SDS-PAGE and Western blot analyses with anti-RGS-His and anti-GST antibodies revealed protein bands with apparent molecular weights consistent with the calculated molecular weights of the *S. macrospora* CAs (data not shown).

When comparing the entire amino acid (aa) sequence of the β-CAs of *S. macrospora* and *N. crassa*, all enzymes shared a high degree of aa sequence similarities. BLASTP analysis of the identified proteins also revealed high degrees of sequence similarities to a recently characterized fungal β-CA from the pathogenic yeast *C. albicans*, two CAs of the pathogenic basidiomycete *Cryptococcus neoformans* as well as to non-characterized homologues from other ascomycetes and the zygomycete *Rhizopus oryzae* (Figure 1A+B).

### Expression of *cas* genes in *S. macrospora*

**Figure 2:**
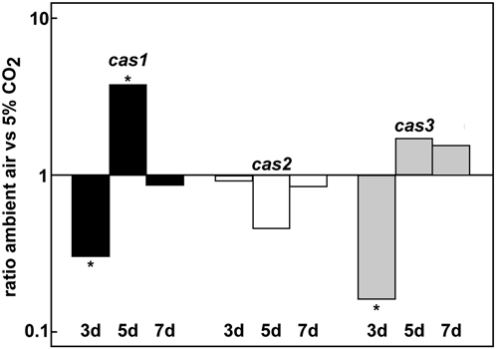
Expression analyses of *cas* genes. Real time PCR was performed with total RNA isolated from *S. macrospora* wild type grown at 27°C in liquid BMM for three, five and seven days under ambient air conditions or 5% CO_2_. Comparisons are given as logarithmic values of the ambient air/5% CO_2_ ratios and are mean expression ratios from two independent biological replicates, each done in triplicate. For normalization, transcript levels of the SSUrRNA were calculated as described in Material and Methods. Asterisk indicate significance according to REST [70].

The CA Nce103p of *S. cerevisiae* is induced by low CO_2_ levels, whereas *C. albicans* Nce103p is constitutively expressed [12], [13]. To understand the functions of CAs in the filamentous fungus *S. macrospora*, we investigated the transcriptional expression of *cas1*, *cas2* and *cas3* in the wild type grown in ambient air or with exposure to 5% CO_2_, by real time PCR. In all experiments, *cas1* mRNA levels were significantly lower under ambient air conditions after three days of development and up-regulated after five days whereas *cas2* transcript was not significantly regulated under the conditions investigated. Expression of *cas3* was significantly down-regulated under ambient air conditions after three days of growth and not regulated by different CO_2_ conditions after five and seven days (Figure 2).

### Subcellular distribution of carbonic anhydrases

**Figure 3:**
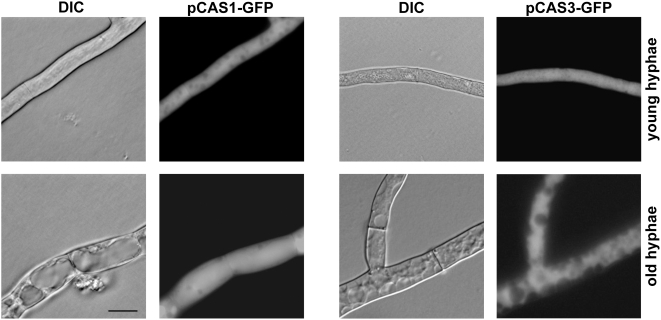
Fluorescence microscopic analyses of *S. macrospora* wild type strains expressing *cas1-egfp* or *cas3-egfp*. The images illustrate the fluorescence of CAS1-EGFP and CAS3-EGFP caused by the transformation of plasmids pGFP-CAS1 or pGFP-CAS3, respectively. Transformants were analyzed after growth for two days on solid SWG medium supplemented with hygromycin. DIC: differential interference contrast. Scale bar indicates 20 µm.

**Figure 4:**
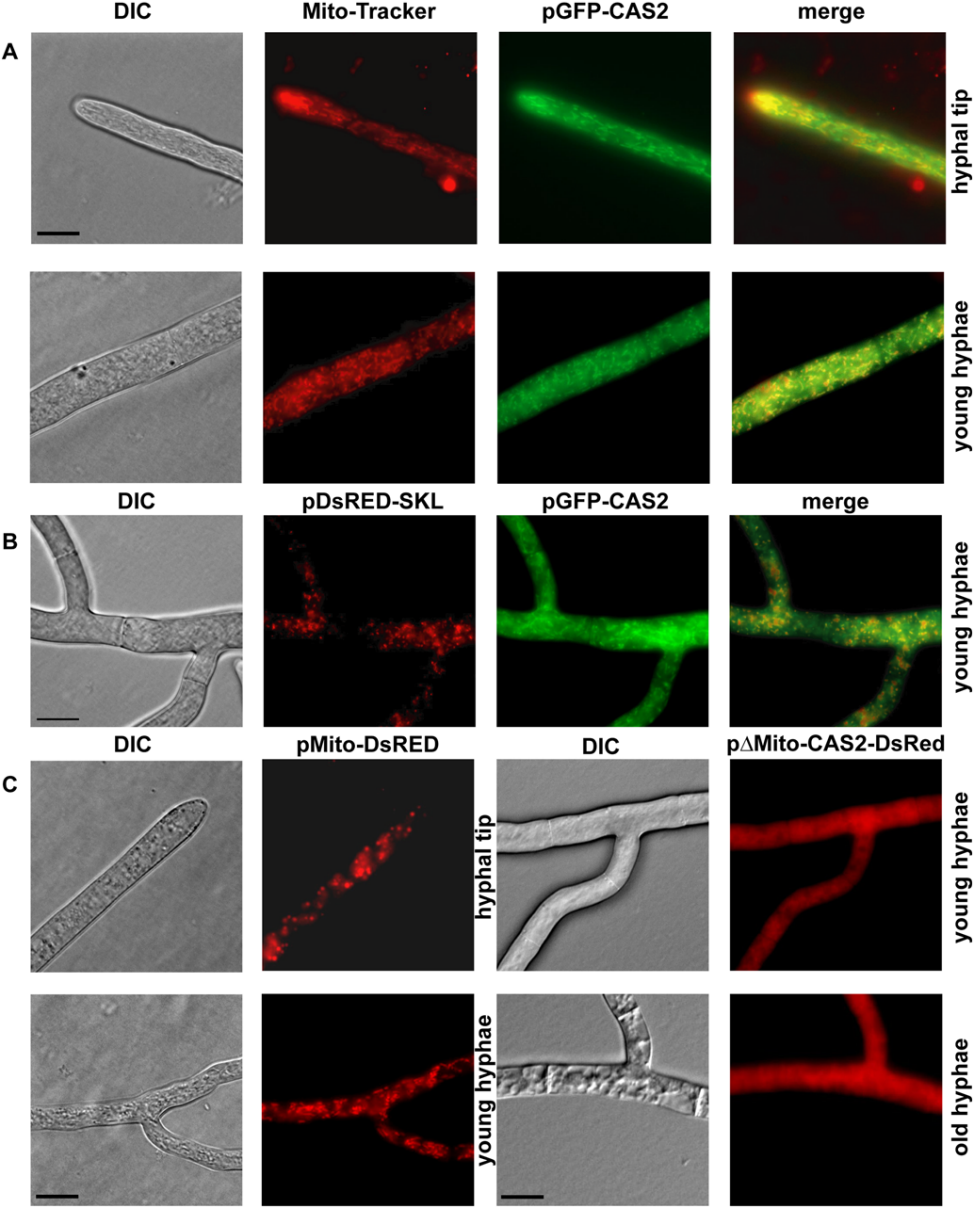
Localization of the carbonic anhydrase CAS2. (A) Fluorescence microscopic analysis of a *S. macrospora* wild type strain carrying plasmid pGFP-CAS2. Expression of full-length *cas2-egfp* leads to EGFP import into mitochondria, visible as tubular structures. CAS2-EGFP co-localizes to mitochondria stained with Mito-Tracker (Invitrogen, Germany) (B) Co-transformation of pGFP-CAS2 and pDsRED-SKL results in clearly distinguishable signals from green fluorescent mitochondria and red peroxisomes. (C) Fluorescence microscopic analysis of a *S. macrospora* wild type strain carrying either pMito-DsRED (N-terminal mitochondrial signal sequence of *cas2* fused to *DsRed*) or pΔMito-CAS-DsRED (*cas2* gene without the signal sequence fused to *DsRed).* DIC: differential interference contrast. Scale bar indicates 20 µm.

To gain a better understanding of the cellular localization of CAs in *S. macrospora*, we performed fluorescence microscopy experiments and transformed plasmids pCAS1-GFP, pCAS2-GFP and pCAS3-GFP carrying *cas-egfp* fusion genes under control of the strong constitutive *gpd* promoter of *A. nidulans*, into the *S. macrospora* wild type. According to an *in silico* TargetP and MitoProt II - v1.101 analysis, CAS2 was predicted to be located in the mitochondria, whereas CAS1 and CAS3 were predicted to be cytoplasmic. Microscopic analysis of the transformants revealed that CAS1-EGFP and CAS3-EGFP proteins were uniformly distributed throughout the cytoplasm of fast-growing, young hyphae, as well as old hyphae. Fluorescence was not concentrated in any organelle and was absent from vacuoles (Figure 3). The CAS2-EGFP fusion protein clearly localized to tubular structures throughout young hyphae and hyphal tips (Figure 4A). Tubular or round-shaped structures are typical for mitochondria in fungal hyphae and a high density of mitochondria can be often found at hyphal tips [26].

To confirm the mitochondrial localization of CAS2-EGFP, we stained the hyphae with Mito-Tracker or, as a negative control; we performed a co-transformation of pCAS2-GFP and pDsRED-SKL. Plasmid pDsRED-SKL encodes a DsRED with a classical C-terminal PTS1 signal for peroxisomal matrix import [27], [28]. The CAS2-EGFP fusion protein clearly co-localized with the Mito-Tracker, indicating that CAS2 is a mitochondrial protein (Figure 4A). No co-localization was observed with DsRED-SKL. Co-transformants carrying pDsRED-SKL and pCAS2-GFP displayed a punctuated red fluorescence pattern of the peroxisomes that did not overlap with the green fluorescent tubular structures of mitochondria (Figure 4B). Some yellow patches in the merge might be because the fluorescent proteins, which were present in the specific organelles, were located in different planes. Overall, the subcellular distribution of CAS2-EGFP was clearly different from that of the DsRED-SKL marker protein.

To address the question of whether the predicted mitochondrial target sequence is sufficient for translocation into mitochondria, we constructed plasmids pMito-DsRED and pΔMito-CAS2-DsRED. According to MitoProt II-v1.101 prediction program [29], CAS2 should be targeted to mitochondria with a probability of 98%. The N-terminus is predicted to be cleaved between residue His59 and Ser60. Hence, we fused the coding region of the 60 N-terminal residues of CAS2 to *DsRed* and analyzed the subcellular distribution of the fusion protein after transformation of pMito-DsRED into the *S. macrospora* wild type. As expected, this protein also localized to tubular or punctate structures, indicating its transport into mitochondria (Figure 4C). Furthermore the expression of *cas2* without its mitochondrial target sequence, encoded by plasmid pΔMito-CAS2-DsRED, led to fluorescence appearing uniformly distributed throughout the cytoplasm of the hyphae (Figure 4C). This result showed that the N-terminal 60 amino acid residues of CAS2 were essential and sufficient for correct localization of the CA to mitochondria.

### Generation of *cas* knock-out strains

**Figure 5:**
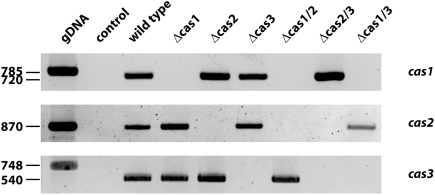
RT-PCR analyses of wild type, single and double knock-out strains to confirm gene deletions. The coding sequence of the *cas* genes were amplified with primer combinations cynT1-GFP-f/cynT1-r, cynT2-GFP-f/cynT2-r and cynT3-GFP-f/cynT3-r, respectively. Equal concentrations of DNA were loaded in each lane. Sizes of amplicons from genomic DNA control (gDNA) and cDNA is indicated aside. Control: negative control, without DNA template.

For a functional characterization of the *S. macrospora* CAs, we generated Δcas1, Δcas2 and Δcas3 deletion strains by gene replacement. Linear fragments containing flanking regions of the desired *S. macrospora cas* gene interrupted by the *hph*-cassette were amplified by PCR from plasmids pCAS1-KO, pCAS2-KO and pCAS3-KO. In the Δcas1 and Δcas2 knock-out constructs, the selectable marker cassette replaced the central part of the gene, including all functional domains, while in the Δcas3 knock-out construct, the complete 5′ region of the gene including all regions encoding putative functional domains was deleted (Figure S1A). Linear knock-out fragments were transformed into the *S. macrospora* Δku70 strain, which is impaired in the repair of DNA double-strand breaks and has been shown to be an ideal recipient strain for gene targeting experiments [30]. Since *S. macrospora* primary transformants are often heterokaryotic, we isolated single spore isolates to segregate the wild-type and mutant alleles of *cas* genes in a Δku70 background. Strains that were homokaryotic for the desired *cas* deletion and carried the *ku70*-deletion were further crossed against color spore mutant fus1-1. By conventional genetic analyses, we succeeded in isolating hygromycin resistant spores without the Δku70 (nourseothricin-resistant) background, indicating that *cas* genes are not coupled to *ku70* and that none of the *cas* genes is an essential gene. PCR amplification with primer pairs specific for external flanking regions in combination with primers specific for the integrated *hph*-cassette confirmed the integration of the hygromycin-resistant cassette at the desired *cas*-locus (Figure S1). By conventional crossing of the single mutant strains, we generated all three possible double knock-out mutants. The genotype of double knock-outs was confirmed by PCR (Figure S1B). In addition, using primer pairs cynT1-GFP-f/cynT1-r, cynT2-GFP-f/cynT2-r and cynT3-GFP-f/cynT3-r, we verified by RT-PCR that transcripts of the deleted genes were absent in all single and double knock-out mutant strains (Figure 5).

### Phenotypic characterization of carbonic anhydrase mutants

**Figure 6:**
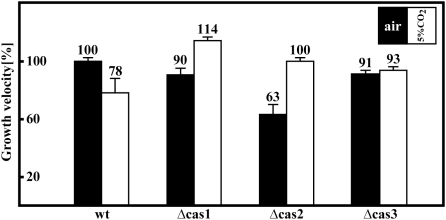
Vegetative growth rate of *S. macrospora* wild type and *cas* mutant strains in ambient air and at elevated CO_2_. Strains were grown for 3 days on solid BMM either in ambient air (black bars) or at 5% CO_2_ (white bars). Growth rate of wild type in ambient air was defined as 100%. Growth rates shown are averages from nine measurements of three independent experiments. Error bars are given as indicated.

**Figure 7:**
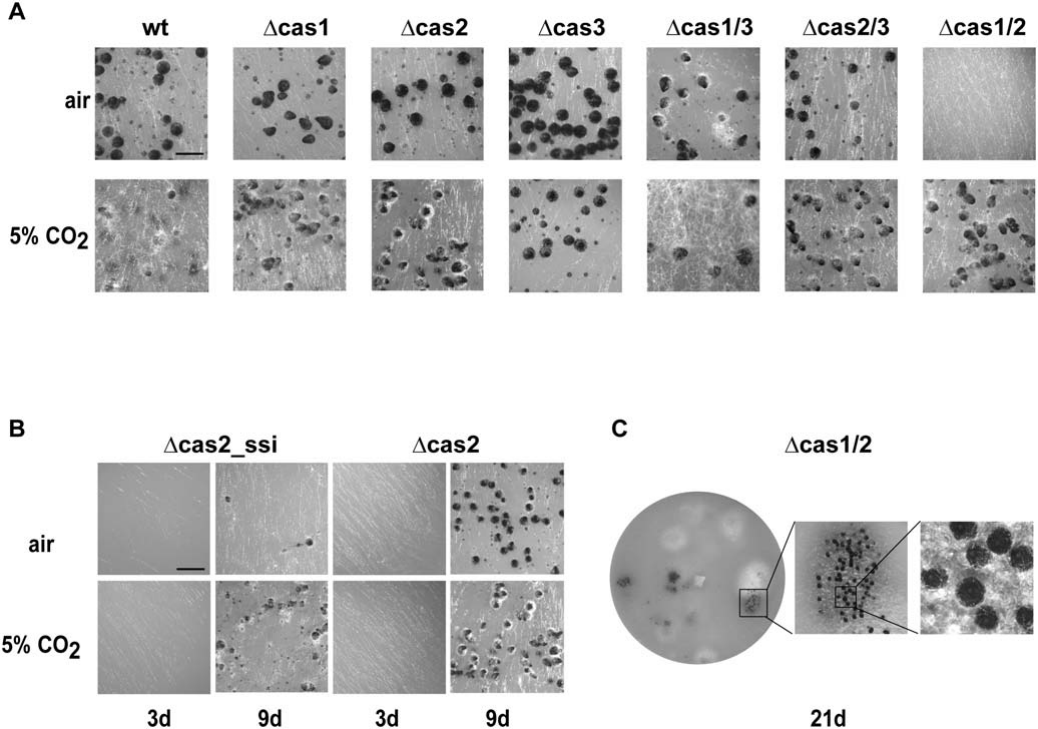
Fruiting-body developmental of wild type, single and double knock-out strains. (A) Strains were grown on BMM medium for 7 days under natural environmental conditions (low CO_2_) or at high CO_2_ concentrations (5%). No morphological changes were observed in single and double Δcas1/3 and Δcas2/3 knock-out strains, respectively. Mature perithecia of Δcas1/2 strain in ambient air were not detectable after 7 days. Elevated CO_2_ concentrations complemented this phenotype. Scale bar indicates 500 µm (B) CO_2_/HCO_3_^−^ accumulates in the fungal cell. Growth defect of Δcas2 is severely increased when mycelium is grown from germinated single spores (Δcas2_ssi: single spore isolate). BMM plate supplied with 0.5% sodium acetate was either inoculated with Δcas2 mutant strain or with an isolated single ascospore of Δcas2. Plates were incubated with or without CO_2_ for three days and images were taken from young mycelia. After prolonged incubation, Δcas2_ssi develops less fruiting-bodies under ambient air than a consecutively transferred mutant strain after nine days of growth. The defect is complemented at 5% CO_2_. Scale bar indicates 1 mm (C) Double knock-out mutant strain Δcas1/2 produced only few perithecia after 21 days of growth. Fruiting-bodies exhibit a wild-type phenotype, but are accumulated at several places on the plate. No mature ascospores were discharged from perithecia developed from Δcas1/2 strain.

The characterization of Δcas1 and Δcas3 single knock-out mutants revealed no obvious phenotypic changes compared to the wild type. Almost no alterations in vegetative growth behavior were detectable, when measuring the growth rate under ambient air conditions (Figure 6). Only Δcas1 showed a 14% elevated growth velocity at 5% CO_2_. Furthermore, neither Δcas1 nor Δcas3 displayed irregularities in mycelial morphology or the formation of sexual reproductive structures (Figure 7). When compared to the wild type or single knock-out strains, the Δcas1/3 double deletion strain also showed no changes in vegetative morphology or reproductive structures. Interestingly, similar to Δcas1, Δcas3 and Δcas1/3 sexual reproduction of the *S. macrospora* wild type was not affected by high CO_2_ concentrations, as recently reported for the basidiomycete *C. neoformans* [16].

In contrast to Δcas1 and Δcas3, the mycelial growth rate of Δcas2 was reduced by 37% under ambient air conditions. This growth defect was fully restored by incubating the mutant strain under elevated CO_2_ conditions (Figure 6). The vegetative growth defect was not rescued by the addition of NaHCO_3_ under ambient air conditions (data not shown). In addition to the reduced growth rate, Δcas2 exhibited a delay in fruiting-body production. While the wild type develops mature perithecia with asci and ascospores after seven days grown on solid corn meal medium under ambient air conditions, the number of fruiting-bodies was slightly reduced in the Δcas2 strain. This defect was also rescued by incubation in high CO_2_ (Figure 7A). Interestingly, both defects of Δcas2, the vegetative growth defect and the reduced number of perithecia, were more evident when the mycelium analyzed was derived from a single germinated Δcas2 ascospore (Figure 7B). Mycelia derived from single spores showed only faint hyphae after three days of growth under ambient air conditions, but this defect was complemented in 5% CO_2_. Furthermore, an apparent decline in the number of mature perithecia was clearly visible when mycelia derived from a single germinated Δcas2 ascospore were grown for nine days in ambient air (Figure 7B). After consecutive transfers of the mycelium from the single spore isolate, this marked effect was reduced and only a slight growth reduction was detectable when compared to the wild type (Figure 6). Thus, CO_2_ and/or HCO_3_^−^ seem to accumulate in the hyphae but not in the ascospore. The phenotypic characteristics of the Δcas2 were also present in the Δcas2/3 double knock-out strain (Figure 7, data not shown). However, in the Δcas1/2 double knock-out strain, fruiting-body development was drastically inhibited under ambient air conditions (Figure 7A). The double mutant produced very few fruiting-bodies after 21 days of growth, and they accumulated only on a few distinct positions of the plate (Figure 7C). Moreover, fruiting-bodies made by Δcas1/2 under ambient air conditions never produced mature ascospores. Only under elevated CO_2_ conditions, after 7–10 days, was the Δcas1/2 mutant able to form a few fruiting-bodies, and then only 75% of these generated a small number of mature ascospores. While perithecia produced by *S. macrospora* wild type contain 105.2±19.9 asci, only 21.1±12.8 asci per perithecium were produced in the Δcas1/2 mutant strain.

RT-PCR analyses of *cas* gene expression in single and double knock-out strains revealed that *cas1* was significantly down-regulated in Δcas3 after five days of growth under ambient air conditions, but not in Δcas2. A slight down-regulation of *cas1* was also obvious in Δcas2/3. The *cas2* transcript was also significantly reduced in the Δcas3 knockout strain, whereas a de-regulation of the transcriptional *cas2* expression was measured in the Δcas1 and Δcas1/3 strains. In contrast, the *cas3* transcript showed no up- or down-regulation in Δcas1, Δcas2 or Δcas1/2 after five days of growth (data not shown).

Since CA mutants of *S. cerevisiae* are more sensitive to several stress-inducing conditions [14], we tested growth and sexual development of wild type and mutant strains on stress inducing media. Neither hyperosmotic media, nor H_2_O_2_, which induces oxidative stress, nor sodium dodecylsulphate, which induces cell-wall damage, led to marked phenotypic differences between wild type and mutant strains (data not shown).

### CAS2 is needed for proper ascospore germination

**Figure 8:**
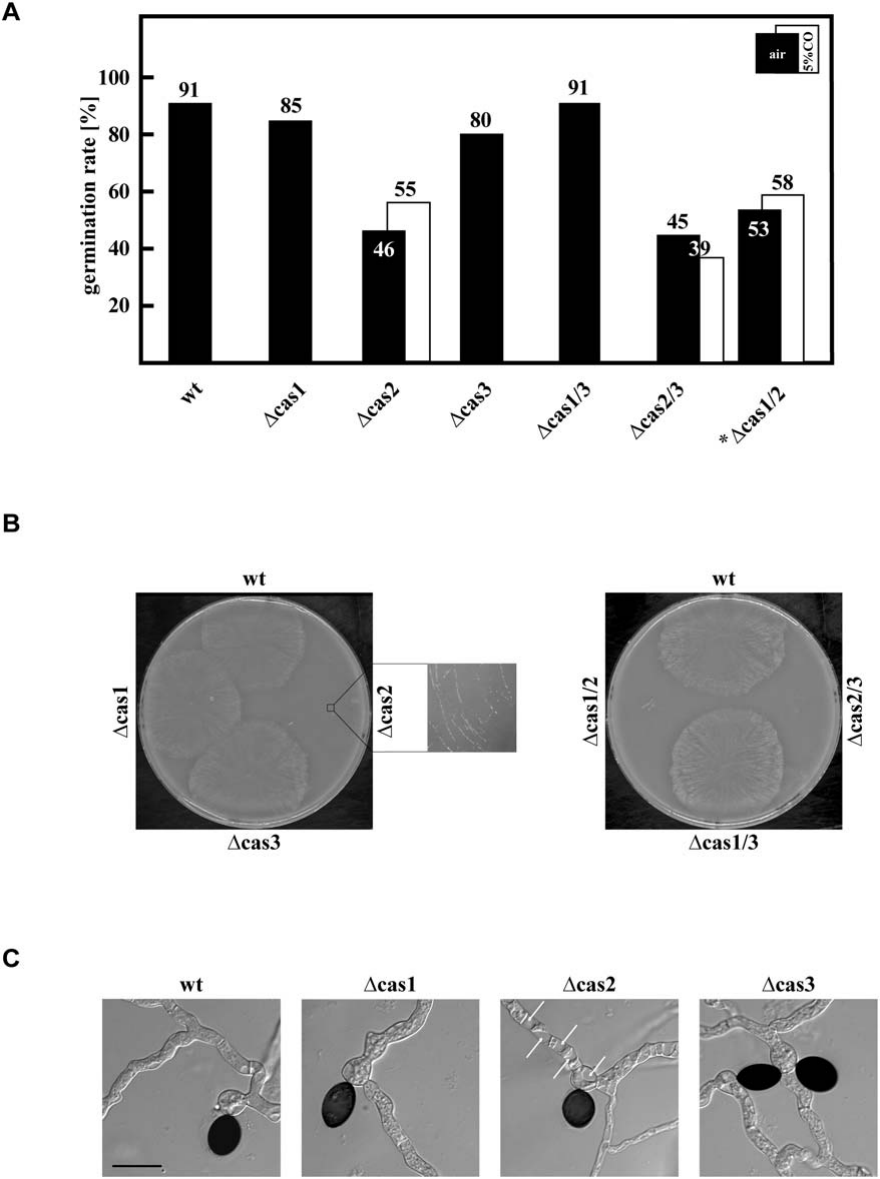
Germination rate of *S. macrospora* wild type and *cas* mutant strains. (A) Ascospores were isolated from strains grown in ambient air. Only strain Δcas1/2 grew at 5% CO_2_ (marked by an asterisk); because it develops mature perithecia with asci and ascospores only at elevated CO_2_ levels (Figure 7). 100 single spores from 10 different perithecia of wild type and mutant strains were isolated and plated on BMM medium with 0.5% sodium acetate. Ascospores were incubated in ambient air or at 5% CO_2_. Germinated spores were counted after 2–5 days of incubation. The percentage of germination efficiency was determined for each strain. (B) Mycelia from germinated spores of Δcas2, Δcas1/2 and Δcas2/3 are affected in growth velocity and hyphal density. Ten spores were plated from each strain and images were taken after two days of growth. (C) Germlings of Δcas2 mutants are highly vacuolated. Spores were plated on glass slides overlaid with a thin layer of BMM supplied with 0.5% sodium acetate and incubated for one day. Vacuoles in hyphae of Δcas2 are marked by white arrows Scale bar indicates 50 µm.

To assess the role of β-CAs during ascospore germination, we investigated the ascospore-germination rate of single and double mutants (Figure 8a). Under ambient air conditions, the germination efficiency of Δcas2 and Δcas2/3 decreased by about 50% while all other mutants exhibited germination efficiencies similar to wild type. Interestingly, the spore germination defect of Δcas2 and Δcas2/3 was not complemented by 5% CO_2_ concentration. The Δcas1/2 strain produced ascospores only when grown at 5% CO_2_, therefore we isolated ascospores from fruiting-bodies developed under this condition. These exhibited a germination defect similar to the Δcas2 single and the Δcas2/3 double knock-out strain. Compared to wild type, the germination efficiency of Δcas1/2 decreased to 58% when spores were germinated under ambient air conditions, and to 53% when spores were germinated under elevated CO_2_ (Figure 8A). As described for Δcas2 and Δcas2/3, mycelia derived from ascospores of Δcas1/2 showed a decreased growth velocity and hyphal density under ambient air conditions (Figure 8B). In contrast, no defects in mycelial growth were observed when spores were germinated at 5% CO_2_ (data not shown). The severe growth defect of Δcas2 single and double knockout mutant strains was also seen when ascospore germination was investigated under the microscope (Figure 8c). While wild type, Δcas1 and Δcas3 ascospores form non-vacuolated hyphae, germlings of the Δcas2 mutant exhibited multiple vacuoles.

### Complementation of Δcas1/2-fus strain by overexpression of *cas1*, *cas2* and *cas3*

To address the question whether the overexpression of one of the β-CAs of *S. macrospora* can complement the severe developmental defects in the Δcas1/2 double mutant, we transformed this strain with overexpression constructs of *cas1*, *cas2*, *cas3* and *cas2*-CTG. The latter construct contains a variant of *cas2* that lacks the mitochondrial target sequence and starts with a CTG codon instead of an ATG. This construct was made because the *N. crassa* homologue of *cas2*, ORF *NCU08133.3*, is annotated with a CTG start codon. The CTG is conserved in the predicted *S. macrospora cas2* ORF, but if the homologous CTG was used as a start codon, the CAS2 protein would lack 35 N-terminal residues and have a truncated mitochondrial target sequence.

**Figure 9:**
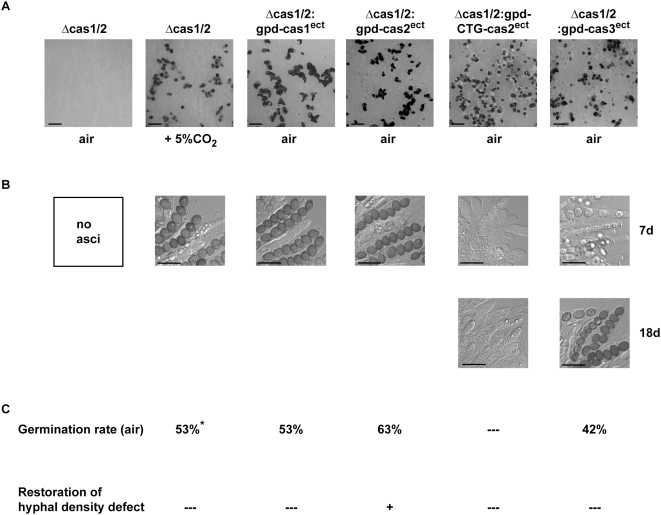
Complementation of the mutant phenotype of Δcas1/2 by ectopically integrated *cas1*, *cas2* and *cas3*, respectively. (A) Morphological characterization of Δcas1/2 strain complemented with different *cas* genes. The images show vegetative hyphae in case of Δcas1/2 strain grown in ambient air and fruiting-body development when grown at 5% CO_2_ or complemented with *cas1*, *cas2*, or *cas3*. The gene *cas2* starts either with an ATG or CTG start codon; all constructs are driven by the constitutive *gpd* promoter of *A. nidulans*. Scale bar indicates 1 mm (B) At ambient air condition, mature ascospores were produced and discharged, only when Δcas1/2 is grown at elevated CO_2_-levels or complemented either with *cas1* or ATG-*cas2*. After a prolonged incubation time of 18 days, complementation with *cas3* also results in discharge of few mature ascospores. Scale bar indicates 50 µm (C) Germination rate and restoration of vegetative growth defects is partially complemented by constitutively expressed *cas2* starting with ATG (+), but not by *cas1*, *cas2*-CTG and *cas3* (—). Asterisk indicates that spores were isolated from a strain grown at 5% CO_2_, because no ascospores were developed in ambient air.

In all transformants, the *cas* gene was ectopically integrated and under control of the strong constitutive *gpd* promoter of *A*. *nidulans*. Overexpression of an ectopic *cas2* did almost fully complement the mutant phenotype of Δcas1/2 (strain Δcas1/2-gpd-cas2^ect^). Mature perithecia with a wild type-like number of asci developed, and mycelial colonies exhibited a wild type-like hyphal morphology as well as a wild type-like colony density. However, the number of germinated ascospores (63%) was only slightly increased in comparison to the mutant strain (53%) (Figure 9). Thus, it seems that only transcription from the endogenous *cas2* promoter led to a complementation of all developmental defects. Overexpression of *cas2*-CTG complemented the defects of Δcas1/2 even worse. Transformants carrying pGPD-CAS2-CTG develop fruiting-bodies, but discharge no mature ascospores. Cracking of the fruiting-bodies revealed the presence of only undifferentiated asci (Figure 9B). This result indicated that *S. macrospora* is able to translate a *cas2*-CTG ORF, but that the truncated CAS2 is not able to fully complement sterility of Δcas1/2. To investigate whether full complementation depends on translocation of CAS2 into the mitochondria, we tested whether the truncated mitochondrial target sequence encoded by pCTG-CAS2-DsRED can target DsRED into mitochondria. For this experiment, plasmid pCTG-CAS2-DsRED was transformed into a *S. macrospora* wild type strain. As shown in Figure S2, the CTG-CAS2-DsRED fusion construct was mainly distributed throughout the cytoplasm of the hyphae, and in only a few hyphal tips were fluorescent tubular structures detectable, indicating that the construct was inefficiently targeted to mitochondria. This result shows that both CAS2 variants may be produced *in vivo*, but that only the full length version of the *cas2* is able to fulfill its complete function.

In contrast to the truncated version of *cas2*, overexpression of *cas1* lead to the formation of fertile fruiting-bodies with asci and mature ascospores under ambient air, whereas overexpression of *cas3* resulted in a reduced number of asci and ascospores. Moreover, these generated only after a prolonged incubation of 18 days (Figure 9B). Determination of the ascospore germination rate of Δcas1/2 strains overexpressing *cas1* or *cas3* resulted in a decreased germination efficiency, which showed that the germination defect was neither complemented by *cas1* nor by *cas3* (Figure 9C).

### Complementation of Δcas1/2-fus strain by the addition of fatty acids

Since HCO_3_^−^ is an important key substrate for decarboxylating enzymes involved in various biosynthetic pathways, it has been hypothesized that the lack of CA activity might affect the acetyl-CoA carboxylase dependent biosynthesis of fatty acids [16], [31]. Therefore, we tried to complement the sterile mutant strain Δcas1/2-fus by the addition of a mixture of fatty acids (palmitic, myristic and stearic acid and 1% Tween 80 as surfactant) in different concentrations. Corn meal medium supplemented with 15 or 30 mg/l of fatty acids completely restored the vegetative growth defect of Δcas1/2-fus, whereas 60 mg/l leads to an increased production of aerial hyphae. Sexual development was slightly revised, because perithecia were formed earlier in contrast to ambient air conditions without fatty acids and they were equally distributed all over the plate (data not shown). Nevertheless, no mature ascospores were produced by the Δcas1/2-fus deletion strain after 14 days of growth under the conditions tested. The Δcas1/2-fus:gpd-cas2^ect^ and wt were not influenced by the addition of fatty acids.

## Discussion

Although the main function of CAs in all organisms is to accomplish the regulation of CO_2_/HCO_3_^−^ homeostasis little is known about how CO_2_ is metabolized and transported in mycelial fungi. Despite being well-characterized in prokaryotes, plants and animals, CAs have been analyzed in only three fungal species [12], [13], [16], [32], [33]. The data presented here demonstrate that three β-CAs are encoded within the genome of the filamentous ascomycete *S. macrospora*. To the best of our knowledge, this is the first genetic analysis on the function of β-CAs in filamentous ascomycetes.

### *S. macrospora* has three β-CAs

The proteins encoded by *cas1*, *cas2* and *cas3* showed a significant degree of sequence identity when compared to known β-CAs of bacterial and plant origin. The crystal structures of β-CAs from plants, bacteria, the archaeon *Methanobacterium thermoautotrophicum* and *C. neoformans* revealed that the active site zinc is coordinated by two conserved cysteines and one conserved histidine [32], [34]–[38]. In addition to these three residues, an aspartic acid and an arginine are also structurally conserved in every β-CA sequence known [4]. These two residues presumably assist in a number of catalytic steps, including substrate binding, proton shuffling and product release, or act as the fourth zinc ligand [32], [35], [39]. Sequence alignment demonstrated that these five residues are also conserved in CAS1, CAS2 and CAS3 (Figure 1A). Based on other key active site residues, β-CAs can be further divided into “plant”-type β-CAs, including all known sequences of plants and of some bacteria and into “cab”-type enzymes of *M*. *thermoautotrophicum*, *Mycobacterium tuberculosis* and *Bacillus subtilis* and other bacteria and archaea [36], [40]. Active site residues corresponding to key active site residues Gln-151, Ser-161, Ser-163, Phe-179, and Tyr-205 of the *Pisum sativum* β-CA [36] are conserved in CAS1 and CAS2. Only Val-184 is replaced by Ile in CAS1 as well as in CAS2. However, a survey of other plant β-CA sequences indicated that either Ile or Val is found at this position [36]. Thus, CAS1 and CAS2 belong to “plant”-type β-CAs. Similar to “cab”-type β-CAs, in CAS3, the amino acid residues corresponding to *P. sativum* Gln-151 are substituted with Pro, Ser-161 with Met, Ser-163 with Ala, Phe-179 with His, Val-184 with Ala and Tyr-205 with Val. Therefore, CAS3 can be classified as a “cab”-type β-CA [36].

A further key difference between CAS1, CAS2 and CAS3 is the N-terminal extension of CAS2, which encodes for a mitochondrial target sequence. This is not a special feature of CAS2 from *S. macrospora*. CAs located in the mitochondria have been identified in algae, plants and animals [6], [8], [41]–[43]. A careful analysis of the complete genome sequences of other filamentous ascomycetes revealed that many of them encode one isoform with a mitochondrial target sequence. These sequences differ in length and exhibit very low sequence identity to the target sequence of *S. macrospora* [44]. The CAS2 homologue of *N. crassa* NCU08133.3 has been annotated without a mitochondrial target sequence and has been predicted to start with a CTG codon, a codon also conserved in *S. macrospora* CAS2. TargetP reliability indicated a lower likelihood of transport of the predicted protein NCU08133.3 into mitochondria. Since expression of a *S. macrospora* CTG variant of *cas2* is not able to fully complement the phenotype of a Δcas1/2 deletion strain, we speculate that the genomic annotation of the *N. crassa* homologue is not correct. Re-annotation of the *N. crassa NCU08133.3* by extending the first exon would lead to a protein with a mitochondrial target sequence [44].

### Transcriptional regulation of β-CA genes in response to CO_2_ differs

Three β-CA genes are simultaneously expressed in *S. macrospora*. *Cas1* and *cas3* are significantly regulated by CO_2_ on day three and five of development, whereas *cas2* seems to be constitutively expressed during development (Figure 2). After three days of growth mRNA levels of *cas1* and *cas3* are significantly lower under ambient air conditions than under elevated CO_2_. While *cas1* becomes up-regulated on day five under ambient air conditions, *cas3* is no longer regulated by CO_2_. This result might implicate a specific function for CAS1 under CO_2_-limited conditions with regard to the production of sexual structures. Examination of the transcriptional regulation of fungal β-CA genes revealed that *Nce103* of *S. cerevisiae* is up-regulated at low CO_2_ levels, whereas expression of *C. albicans Nce103* and *C. neoformans CAN2*, encoding the major CA of *C. neoformans*, are not regulated by the CO_2_ concentration [12], [16], [45], [46].

Evidence for a possible dependency between *cas1* and *cas2* is their transcriptional regulation in different *cas* knockout backgrounds. Whereas *cas1* is not significantly regulated in a Δcas2 background, *cas2* is completely de-regulated in Δcas1 and Δcas1/3 deletion strains. Although the Δcas1 strains do not exhibit any morphological phenotype, it seems that *cas2* must be antagonistic to *cas1*.

Similar to *cas1*, *cas3* is only down-regulated at day three of sexual development but constitutively expressed under both conditions at later stages of development. However, in contrast to Δcas1 and Δcas2, growth of a Δcas3 strain is not influenced by elevated CO_2_ conditions (Figure 6). Interestingly, the transcriptional regulation is also not influenced by the presence or absence of the other two CA genes, indicating that *cas3* might be involved in another bicarbonate dependent pathway that is independent from CO_2_ concentrations under the conditions tested.

### CAS2 is targeted to mitochondria and is required for germination and vegetative growth

In many eukaryotes encoding multiple CAs, different isoforms of the enzyme have been shown to be distributed in different tissues and cell compartments [3]. When fused to *egfp*, the product of *cas2* was exclusively targeted to the mitochondria, thus confirming the *in silico* prediction of a classical mitochondrial import sequence by TargetP and MitoProt II. While α-, β- and γ-CAs have been reported to become targeted to mitochondria in mammals, plants and algae [3], [6], [8], [42], this is the first time that a fungal β-CA has been shown to be located exclusively to mitochondria.

No rigorous phenotypic changes related to sexual development were observed in CA single knockout strains. Only the Δcas2 mutant displayed a weak delay in vegetative growth, and this defect was complemented by the elevation of the CO_2_ concentration. Deletion of the *cas2* gene may lead to a decrease in the mitochondrial bicarbonate concentration under ambient air conditions.

In algae mitochondrial CAs are involved in the inorganic carbon-concentrating mechanism, supporting anaplerotic β-carboxylation and they are proposed to act as pH stabilizers [42], [47], [48]. Raven [49] hypothesized that if there is a HCO_3_^−^ channel in the inner mitochondrial membrane, almost all of the inorganic carbon would leave the mitochondria as HCO_3_^−^, thus limiting the potential for CO_2_ leakage through the plasmalemma. Similarly, mitochondrial β-CA CAS2 of *S. macrospora* may contribute to the cytoplasmic bicarbonate pool required for carboxylating enzymes relevant to many metabolic processes. Among these, acetyl-CoA carboxylase uses bicarbonate to produce malonyl-CoA for fatty acid synthesis. Indeed, it was shown recently that biosynthesis of fatty acids is affected by deletion of fungal CA genes. In the basidiomycete *C. neoformans*, the phenotype of a Δcan2 knockout was partially complemented by the addition of the fatty acid palmitate [16]. The growth defect of a *S. cerevisiae* Δnce103 strain under atmospheric pressure can be complemented only when end-products of bicarbonate-dependent metabolic pathways are supplied to the growth medium [31]. Complementation assays with a mixture of fatty acids also restored the vegetative growth defect of CA-deletion mutants in *S. macrospora*.

In addition to the vegetative growth defect, the Δcas2 strain also displays a considerable decrease in germination efficiency, which was also obvious in Δcas2/3 and Δcas1/2 double deletion strains. *S. macrospora* appears to need a certain HCO_3_^−^ concentration in the mitochondria for efficient ascospore germination. In contrast to vegetative growth defects, this defect of the Δcas2 mutant is not complemented by an elevated CO_2_ concentration. Therefore, we conclude that CAS2-mediated CO_2_/HCO_3_^−^ homeostasis in mitochondria plays an important role in spore germination and in vegetative growth. Interestingly, the vegetative growth defect increased when colonies were derived from germinated spores. The mycelium derived from a germinating Δcas2 ascospore was composed of thin vacuolated hyphae indicating that CAS2 is needed at conditions of higher HCO_3_^−^ demand (Figure 7B). Ascospores are dormant, metabolically-inactive cells, thus CO_2_ production through respiration is rather low and the HCO_3_^−^ level is consequently low as well. We hypothesis that HCO_3_^−^-dependent carboxylation reactions are needed for sufficient nutrient supply and energy recovery of the germinating ascospore. However, after a prolonged time of vegetative growth, HCO_3_^−^ generated by CAS1 and/or CAS3 accumulates in the cell and partially abolishes the growth impairment.

Taking our results into account, CAS3 appears not to be responsible for the HCO_3_^−^ accumulation effect in the Δcas2 mutant strain. Δcas3 exhibited no obvious morphological phenotype under any conditions tested. Furthermore, a Δcas2/3 double knock-out strain behaved similarly to the Δcas2 mutant. HCO_3_^−^ produced by CAS3 might be the co-factor for a reaction that is not linked to sexual reproduction, vegetative growth or ascospore germination. In this context, it would be interesting to investigate a possible connection between CA mediated HCO_3_^−^ production and cyanate decomposition, a reaction that in *S. macrospora*, has been recently shown to be catalyzed by a bicarbonate-dependent cyanase. Deletion of the cyanase-encoding gene *cyn1* does not lead to any defects in vegetative growth or fruiting-body development [50]. In *E. coli* the β-class CA CynT is encoded in an operon together with the cyanase-encoding gene *cynS*, and the function of the *E. coli* CA appears to be to prevent HCO_3_^−^ depletion during cyanate decomposition [51].

### Deletion of *cas1* and *cas2* blocks sexual reproduction

The Δcas1/2 double deletion strain displays a more severe phenotype than the Δcas2 single knockout strain and the Δcas2/3 double knockout strain, indicating that in addition to CAS2, CAS1 is also an active CA. While perithecia produced by *S. macrospora* wild type contain about 100 asci in ambient air [52], the production of fruiting-bodies and differentiated ascus rosettes is drastically impaired in the Δcas1/2 double deletion strain. A small number of fruiting-bodies with only immature asci was produced after 21 days of growth. The defect could be partially complemented by incubation at elevated CO_2_ levels, which resulted in the production of fruiting-bodies containing significantly fewer mature ascospores than the wild type (Figure 7 and Figure 9). The phenotype of the Δcas1/2 mutant resembles the *S. macrospora* developmental mutant per5, which produces only ascus precursors without any mature spores. The per5 mutant has been shown to encode a defective ATP-citrate lyase that is involved in the formation of acetyl-CoA used for the biosynthesis of fatty acids [53]. In contrast to the complementation of the vegetative growth defect, the impaired production of mature asci in the Δcas1/2-fus was not reversed by fatty acids. This result indicates that specific CA activity impacts further biochemical pathways than fatty acid biosynthesis during sexual development. Thus, in 5% CO_2_, the small amount of bicarbonate generated through nonenzymatic, spontaneous hydration of CO_2_ and/or through the activity of the remaining CAS3 is sufficient to support fruiting-body and ascospore formation. Similarly a Δcan2 mutant of *C. neoformans* was shown to exhibit a severe sporulation defect [16]. However, it should be taken into account that complementation of Δcas1/2-fus strain by overexpression of *cas1*, *cas2* and *cas3* due to the use of a strong heterologous *gpdA* promoter of *A. nidulans* could increase some paths and decrease others, consequently affecting the overall phenotype.

These results demonstrated that the protein encoded by *cas3* has CA activity, albeit it is probably less active than CAS1 and CAS2. Growth defects caused by the deletion of CA genes in bacteria, have been shown to be rescued by introducing heterologous CA genes [54], [55]. The growth defect of a *S. cerevisiae* CA-mutant strain was complemented by plant, plant chloroplast, human, prokaryotic and fungal CAs [14], [32], [56], [57]. Apparently, in these unicellular organisms, only a small amount of CA activity is sufficient for growth at ambient air conditions, irrespective of its origin, whereas in *S. macrospora*, mitochondrial targeting of CAS2 seems to also be essential for growth and sexual reproduction.

In summary, complementation assays in combination with phenotypic observations indicate that in *S. macrospora cas2* and possibly also *cas1* may encode for a major CA involved in fruiting-body and ascospore development, as well as in ascospore germination, while *cas3* encodes for a minor CA. This must be confirmed by *in vitro* activity measurements, however, as recently shown for the *C. neoformans* and the *C. albicans* CAs [58]. Furthermore, analysis in *C. albicans* and *C. neoformans* revealed a functional link between cAMP signaling and CO_2_/HCO_3_^−^ sensing and showed that bicarbonate directly activates adenylyl cyclase [7], [13], [15]. Similar to the phenotypes presented here, deletion of adenylyl cyclase in *S. macrospora* has recently been shown to affect vegetative growth and sexual development in *S. macrospora* [19]. In this respect, further examination of the functional connection between CAs and adenylyl cyclase in fruiting-body and ascospore formation of filamentous ascomycetes will be of great interest.

## Materials and Methods

### Strains, media and culture conditions

Cloning and propagation of recombinant plasmids was done under standard conditions with *Escherichia coli* strain SURE used as a host for plasmid amplification [59]. Genotypes of *S. macrospora* strains used and generated in this study are provided in Table 1. All strains were cultivated either on solid or in liquid corn meal medium (BMM) or in liquid complete medium (CM) or on solid SWG medium [23], incubated in ambient air or at 5% carbon dioxide in the Inkubator C42 (Labotect, Germany). Transformation of *S. macrospora* was performed as described previously with 20 mg/ml Glucanex 200G (Schliessmann, Germany) to degrade fungal cell walls [53]. The determination of germination rates were conducted by isolating 100 single ascospores. After inoculation for 2–5 days on BMM supplied with 0.5% of sodium acetetate, the number of germinated spores was determined. Dry cell weight was measured according to Nolting and Pöggeler [60] and growth velocities were either determined according to Nowrousian and Cebula [61], or by measuring the colony radius on agar plates after 1 to 10 days. For complementation assays with fatty acids, 30 mg/l of palmitic, myristic and stearic acid and 1% Tween 80 were added to BMM medium according to Aguilera et al. [31]. Genomic DNA was isolated from cultures grown for 3 days in liquid CM medium; RNA samples for qRT-PCR were isolated from cultures grown for 5 days in liquid CM or BMM.

**Table 1:** *Sordaria macropsora* strains used in this study

Strain	Genotype and Phenotype^1^	Source
S17736	Wild type	Lab Collection^2^
S23442	fus1-1, spore color mutant	Lab Collection^2^
S66001	Δku70::*nat* ^R^ *hph* ^S^	[30]
Δcas1	Δcas1::*hph* ^R^, single spore isolate, fertile	This study
Δcas1-fus	Δcas1:: *hph* ^R^/fus1-1, single spore isolate, fertile	This study
Δcas2	Δcas2::*hph* ^R^, single spore isolate, fertile	This study
Δcas2-fus	Δcas2:: *hph* ^R^/fus1-1, single spore isolate, fertile	This study
Δcas3	Δcas3::*hph* ^R^, single spore isolate, fertile	This study
Δcas3-fus	Δcas3:: *hph* ^R^/fus1-1, single spore isolate, fertile	This study
Δcas1/2	Δcas1::*hph* ^R^/Δcas2::*hph* ^R^ , single spore isolate, sterile	This study
Δcas1/2-fus	Δcas1::*hph* ^R^/Δcas2::*hph* ^R^/fus1-1, single spore isolate, sterile	This study
Δcas2/3	Δcas2::*hph* ^R^/Δcas3::*hph* ^R^, single spore isolate, fertile	This study
Δcas1/3	Δcas1::*hph* ^R^/Δcas3::*hph* ^R^, single spore isolate, fertile	This study
Δcas1/2-fus:gpd-cas1^ect^	Δcas1::*hph* ^R^/Δcas2::*hph* ^R^/fus1-1, ectopic copy of *cas1* under control of *A. nidulans gpd* promoter, *nat* ^R^ , single spore isolate, fertile	This study
Δcas1/2-fus:gpd-cas2^ect^	Δcas1::*hph* ^R^/Δcas2::*hph* ^R^/fus1-1, ectopic copy of *cas2* under control of *A. nidulans gpd* promoter, *nat* ^R^ , single spore isolate, fertile	This study
Δcas1/2-fus:gpd-cas3^ect^	Δcas1::*hph* ^R^/Δcas2::*hph* ^R^/fus1-1, ectopic copy of *cas3* under control of *A. nidulans gpd* promoter, *nat* ^R^ , single spore isolate, affected in ascosporogenesis	This study
Δcas1/2-fus:gpd-CTG-cas2^ect^	Δcas1::*hph* ^R^/Δcas2::*hph* ^R^/fus1-1, ectopic copy of *cas2* (deletion of nucleotides 1–108) under control of *A. nidulans gpd* promoter, *nat* ^R^ , primary transformant, blocked in ascosporogenesis	This study
wt_CAS1-GFP	Primary transformant of S17736 with pGFP-CAS1	This study
wt_CAS2-GFP	Primary transformant of S17736 with pGFP-CAS2	This study
wt_CAS3-GFP	Primary transformant of S17736 with pGFP-CAS3	This study
wt_CAS2-GFP:DsRED-SKL	Primary transformant of S17736 with pGFP-CAS2 and pDsRED-SKL	This study
wt_ΔMito-CAS2-DsRED	Primary transformant of S17736 with pΔMito-CAS2-DsRED	This study
wt_Mito-DsRED	S17736 transformed with pMito-DsRED, single spore isolate	This study
wt_CTG-Mito-DsRED	S17736 transformed with pCTG-Mito-DsRED, single spore isolate	This study

1 hyg^R^ – hygromcin resistant, nat^R^ – nourseothricin resistant

2 Department of General and Molecular Botany, Ruhr-University Bochum

### Sequence analysis

Primers were synthesized at MWG Biotech AG (Ebersberg, Germany). DNA sequencing was performed by the sequencing service of the Department of Biochemistry (Ruhr-University of Bochum, Germany) or by the G2L-sequencing service of the Göttinger Genom Labor (Georg-August University of Göttingen, Germany) or by MWG Biotech Customer Service (Ebersberg, Germany).

Molecular weights and isoelectric points of proteins were calculated with programs from the ExPASy Proteomics Server (http://www.expasy.org). Protein sequence alignments were performed using the ClustalX program [62]. Protein and nucleotide sequence data of β-CAs from other organisms were obtained from the public databases at NCBI (http://www.ncbi.nlm.nih.gov/entrez/) or by BLAST searches of the complete sequenced genomes at the Broad Institute (http://www.broad.mit.edu/annotation/fungi/fgi/). Subcellular localization of carbonic anhydrases was predicted with the TargetP program provided on the CBS Prediction Server (http://www.cbs.dtu.dk/services/) and with MitoProt II - v1.101 [29].

### Preparation of nucleic acids

Isolation of genomic DNA from *S. macrospora* was done as described previously [22]. RNA was isolated from mycelial samples, which were three times incubated in liquid nitrogen for 5 sec. Afterwards the frozen sample was supplemented with 1 ml Trizol (Invitrogen) and homogenized two times (2 min, 1000 Hz) using the TissueLyser (Qiagen), followed by centrifugation (10 min, 20°C, 12,000 rpm). Then, 1 ml from the supernatant was mixed with 0.2 ml chloroform and centrifuged again. One volume of isopropanol was then incubated at −80°C supplemented with 500 µl from the supernatant. After precipitation (10 min, 20°C, 12,000 rpm), the pellet was washed with 500 µl 75% ethanol (10 min, 20°C, 12,000 rpm) and dried at room temperature. The precipitated RNA was resolved in 100 µl of sterile DNA free water and tested on a gel, before it was used for cDNA synthesis and qPCR analysis.

### Quantitative real-time PCR

Reverse transcription was done using the Transcriptor High Fidelity cDNA Synthesis Kit (Roche, Germany) as described by the manufacturers. Quantitative RT-PCR was performed as described previously [63]. The qPCR MasterMix Plus for SYBR green Kit (Eurogentec, Belgium) was used for real-time PCR performance carried out with a Mastercycler^®^ ep *realplex* (Eppendorf, Germany). Primer pair CynT1-RT_f/CynT1-RT_r was used to amplify a 133 bp part of the *cas1* gene. Combinations of primers CynT2-RT_f/CynT2-RT_r and CynT3-RT_f/CynT3-RT_r resulted in amplicons of 134 bp in case of *cas2* and 146 bp in case of *cas3*. Primers used are given in Table S1. All real-time experiments were performed at least two times in triplicates with independent biological samples. An amplicon derived from the small-subunit rRNA was used as a reference for normalization (primer pair SSU-f/SSU-r) according to Pöggeler *et al*. [63].

### Identification and isolation of three genes encoding for carbonic anhydrases

Two different strategies were used to isolate *cas* genes encoding for putative carbonic anhydrases in *S. macrospora*. The genes *cas1* and *cas3* and their flanking sequences were in parts or directly amplified from *S. macrospora* genomic DNA using heterologous oligonucleotides derived from sequences of homologous *N. crassa* genes, while an inverse PCR strategy was used in case of the *cas2* gene according to Nowrousian *et al*. [21]. Oligonucleotides used for cloning are given in Table S1. Firstly, primer pair cynT1-a/cynT1-b, whose nucleotide sequences are based on the sequence of the appropriate *N. crassa* homologue, was used to amplify an internal 593-bp *cas1* fragment, followed by the design of *S. macrospora* specific primers cynT1-c1 and cynT1-d. Primer cynT1-c1 was used in combination with the heterologous primer cynT1-79 (based on the sequence of *NCU04779.3* from *N. crassa*) to amplify a fragment of approximately 2800 bp. Only 689 bp downstream of the *cas1* gene were sequenced and used for further analysis. With primer cynT1-d and the heterologous primer cynT1-77 (*NCU04777.3*), a 2,271-bp fragment located upstream of the *cas1* gene was amplified and sequenced. All together, a 3,558 bp part of the genomic sequence including the 770-bp *cas1* coding region with 5′ and 3′ regions was sequenced.

The 586-bp sequence of an internal part of *cas2* was amplified with the heterologous primer pair cynT2-a/cynT2-b. Since, heterologous oligonucleotides designed according to *N. crassa* genes flanking the *cas2* open reading frame in *N. crassa* failed to amplify sequences adjacent to *cas2* of *S. macrospora,* we performed an inverse PCR strategy as described previously [21]. In brief: genomic DNA from *S. macrospora* was digested with restriction endonuclease *Bsp*120I. Afterwards, all fragments were self-ligated using T4-Ligase (Fermentas, Germany) and precipitated. The DNA was then used for inverse PCR with primer pair cynT2-c1/cynT2-d1 with Thermozym-Hotstart Taq-Polymerase (Molzyme, Germany). The resulting PCR fragment was cloned into pDrive cloning vector (Qiagen, Germany) and sequenced by means of primer walking, resulting in a 2,418-bp fragment including the complete 855-bp *cas2* open reading frame and adjacent 5′- and 3′-regions.

In case of *cas3*, a 3,242-bp fragment with the entire protein coding sequence and 5′ and 3′ flanking regions were amplified with the heterologous primer pair T3-1102/T3-1104, based on the sequence of the adjacent gene homologues *NCU01104.3* and *NCU01102.3* of *N. crassa*. The 3,242-bp fragment was cloned into pDrive cloning vector (Qiagen) and sequenced.

### Construction of fluorescence plasmids

For construction of *cas-egfp* fusion constructs under the control of the constitutive *A. nidulans gpd* promoter, the coding regions of *cas2* and *cas3* were amplified by PCR with primer pairs cynT2-GFP-f/cynT2-r and cynT3-GFP-f/cynT3-r (Table S1) from *S. macrospora* genomic DNA. With this PCR, flanking *Nco*I restriction sites were generated on each end. The fragments were subcloned into pDrive vector (Qiagen, Germany). Since the *cas1* gene contains an internal *Nco*I restriction site, a site directed point mutation was introduced by overlap extension PCR with primer pairs cynT1-GFP-f/T1-Mut-r and T1-Mut-f/cynT1-r (Table S1). All PCR reactions were accomplished, using HotStarTaq DNA Polymerase (Qiagen, Germany). After sequencing, *Nco*I restriction resulted in fragments of 773-, 858- and 736-bp, which were cloned into the single *Nco*I site of *egfp* containing vector p1783.1 to create plasmids pGFP-CAS1, pGFP-CAS2 and pGFP-CAS3, respectively. Plasmid p1783.1 [64] contains the *egfp* gene downstream of the *Nco*I restriction site, resulting in CAs fused to the N terminus of EGFP.

Furthermore, a truncated *cas2-DsRed* fusion gene was constructed by PCR using primers cynT2-56f-NcoI and cynT2-r (Table S1). The amplification resulted in the deletion of the first 165 bp encoding for 55 amino acids. The resulting sequence was verified and the *Nco*I fragment was cloned into the single *Nco*I restriction site of *DsRed* expression vector pRHN1 [65]. This vector is termed pΔMito-CAS2-DsRED (Table S2) and allows expression of DsRED fused to CAS2 without its 55 aa N-terminal mitochondrial target sequence but with a newly generated ATG start codon. In a further approach, the first 180 bp encoding the 60 aa of the CAS2 N-terminus including the mitochondrial target sequence were amplified with primer pair cynT2-GFP-f/Mito-r (Table S1) and were also ligated into the *Nco*I site of pRHN1, resulting in plasmid pMito-DsRED (Table S2). This plasmid was used as a template for an inverse PCR based cloning strategy with oligonucleotides CTG-Mito_f and pEHNnat-r as described previously [66], resulting in the truncation of 105 bp of the mitochondrial target sequence. The final plasmid is termed pCTG-CAS2-DsRED (Table S2) and encodes a CAS2-DsRED fusion protein which starts with a CTG codon instead of an ATG start codon and has a truncation of the first 35 aa of the mitochondrial target sequence of CAS2. All fluorescence plasmids were transformed into *S. macrospora* wild type strain to investigate fluorescence patterns.

### Generation and complementation of *S. macrospora* knock-out strains

Knock-out constructs for homologous recombination were produced utilizing homologous recombination in yeast as described previously [67]. The 5′- and 3′-regions of the genes *cas1*, *cas2* and *cas3* were amplified from genomic DNA of *S. macrospora* wild type strain using primer pairs cynT1-5f/cynT1-5r, cynT1-3f/cynT1-3r, cynT2-5f/cynT2-5r, cynT2-3f/cynT2-3r, cynT3-5f/cynT3-5r and cynT3-3f/cynT3-3r (Table S1). The PCR reactions were conduced to add specific overhangs to the 5′- and 3′-regions of the respective *cas* gene, homologous to the *hph*-cassette and the yeast plasmid pRS426, respectively. The *hph*-cassette was generated with primers hph-f and hph-r using pCYN1-KO as a template [50]. In each case, three PCR fragments together with the *Eco*RI/*Xho*I linearized vector pRS426 [68] were co-transformed into yeast strain PJ69-4A [69], where homologous recombination took place. Transformants were selected on SD plates lacking uracil, afterwards plasmid DNA was isolated with the NucleoSpin^®^ Plasmid Kit (Macherey-Nagel, according to Nolting and Pöggeler [20]). To isolate the resulting plasmids pCAS1-KO, pCAS2-KO and pCAS3-KO (Table S2), total DNA was subsequently transformed into *E. coli* strain SURE and plasmid DNA isolation was carried out. The KO-plasmids were used as templates to amplify the CA-*hph* cassettes with oligonucleotide pairs cynT1-5f/cynT1-3r, cynT2-5f/cynT2-3r or cynT3-5f/cynT3-3r (Table S2 and Figure S1). To generate the Δcas1, Δcas2 and Δcas3 mutant strains, the resulting PCR amplicons (*cas1*: 3,619 bp; *cas2*: 3,071 bp; *cas3*: 3,258 bp) were transformed into *S. macrospora* Δku70 which is impaired in heterologous recombination [30]. In each strain the major part of the CA coding region including its putative catalytic domain is substituted against the *hph* gene (Figure S1). Transformants were screened by PCR with primer pairs which are specific for external flanking regions and the integrated *hph*-cassette (Table S1, Figure S1), and with a primer pair specific to the external flanking 5′ region (Table S1, Figure S1). Additionally, to prove that mutant strains do not contain the respective wild type *cas* gene, primer pairs matching the substituted coding region of the *cas* gene were used, and resulted in amplicons only in the wild type and not in the deletion mutants (data not shown). Furthermore RT-PCR experiments were performed to confirm the knock-out mutants on their transcriptional levels using primer pairs cynT1-GFP-f/cynT1-r, cynT2-GFP-f/cynT2-r and cynT3-GFP-f/cynT3-r.

To complement the phenotypes, rescue plasmids pGPD-CAS1, pGPD-CAS2 and pGPD-CAS3 were constructed by amplifying the entire coding regions with primer pairs cynT1-GFP-f/cynT1-BamHI-r, cynT2GFP-f/cynT2-BamHI-r and cynT3GFP-f/cynT3BamHI-r, respectively. Oligonucleotides used generated *Nco*I and *Bam*HI restriction sites. Genomic DNA was used as template and in case of *cas1* (because of its internal *Nco*I restriction site) with plasmid pGFP-CAS1 as template. After subcloning into pDrive (Qiagen, Germany) and sequencing, specific *Nco*I/*Bam*HI fragments were cloned into pEHN-nat1 (Table S2), containing the nourseothricin resistance cassette. In all constructs, the CA gene is under control the constitutive *A. nidulans gpd* promoter and *trpC* terminator. Plasmid pGPD-CAS2-CTG was constructed, by using primers cynT2-CTG-f and cynT2-BamHI-r to generate an amplicon with *Not*I and *Bam*HI restriction sites. After sequencing, a specific *Not*I/*Bam*HI fragment was cloned into pEHN-nat1. In this case, the *cas2* open reading frame starts with a CTG, encoding for Leu37 in the original gene, instead of the ATG start codon.

### Construction of double knock-out strains

Conventional genetic analyses were used to generate double knock-out strains as described previously [23]. Two single knock-out strains were crossed on BMM medium plates and asci from recombinant perithecia were isolated after 7–10 days. For the identification of hybrid perithecia, mutant strains producing black spores were crossed against mutant strains carrying in addition the spore color marker fus1-1. Single spore isolates of selected asci were analyzed by PCR to verify double knock-out strains.

### Microscopic investigations

*S. macrospora* strains were inoculated on glass slides for 1 to 7 days. Slides were overlaid with BMM or SWG minimal medium as described earlier [18]. Light and fluorescence microscopic investigations were carried out according to Elleuche and Pöggeler [28]. Co-localization of mitochondrial localized CAS2-constructs was carried out using Mito-Tracker (Invitrogen, Germany).

## Supporting Information

Figure S1 Knock-out of cas genes
(0.55 MB TIF)

Figure S2 Localization of CTG-CAS2-DsRED
(1.35 MB TIF)

Table S1 Oligonucleotides used in this study
(0.08 MB DOC)

Table S2 Plasmids used in this study
(0.04 MB DOC)
