# Supplementary figures and images for: β-Carbonic Anhydrases Play a Role in Fruiting Body Development and Ascospore Germination in the Filamentous Fungus Sordaria macrospora
Source: PLoS One. 2009 Apr 13;4(4):e5177. doi: 10.1371/journal.pone.0005177 (PMC2664464; doi:10.1371/journal.pone.0005177)

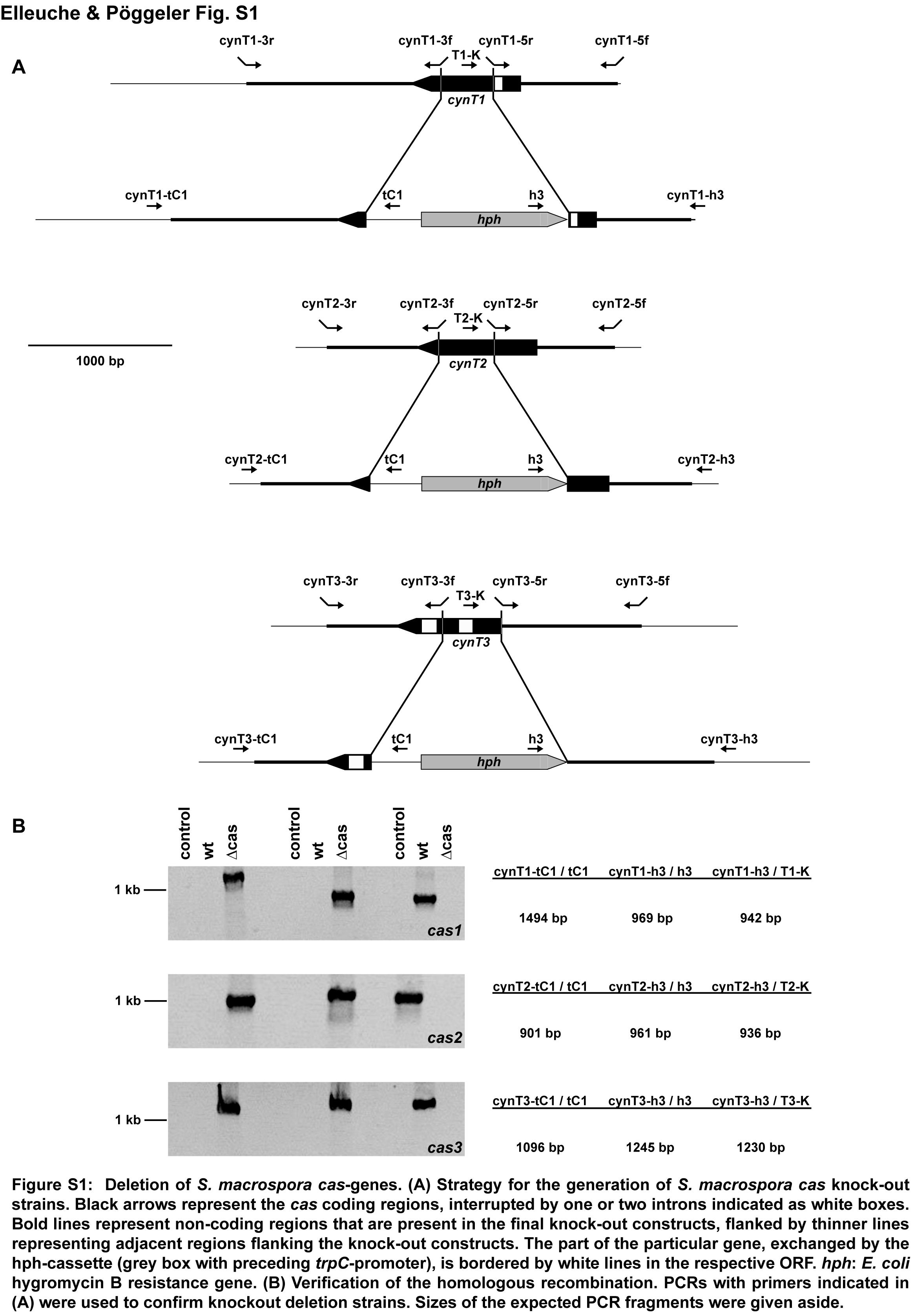

Supplement: Figure S1 — Knock-out of cas genes (0.55 MB TIF) [file pone.0005177.s001.tif]

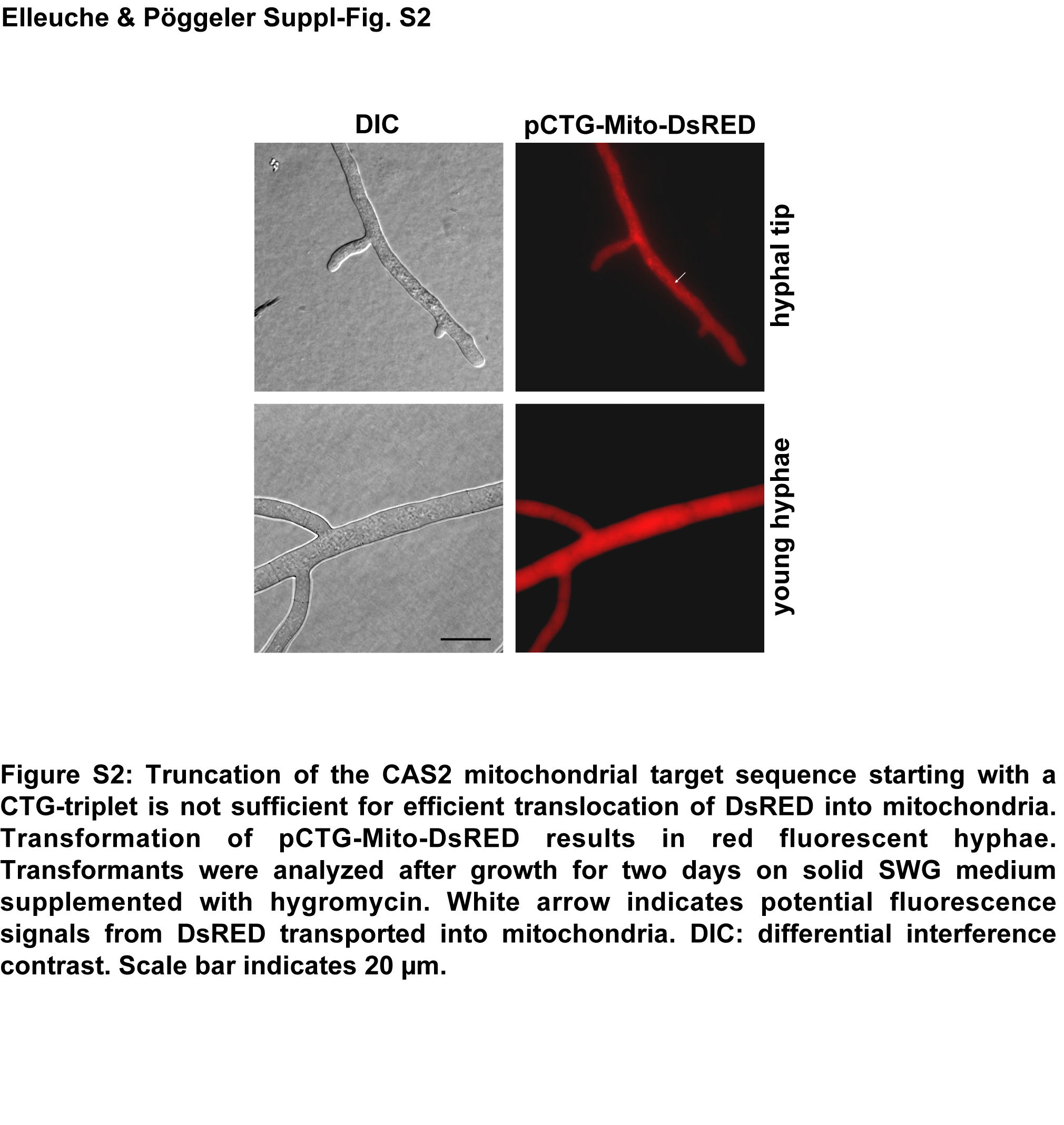

Supplement: Figure S2 — Localization of CTG-CAS2-DsRED (1.35 MB TIF) [file pone.0005177.s002.tif]
