# Supplementary material for: β-Carbonic Anhydrases Play a Role in Fruiting Body Development and Ascospore Germination in the Filamentous Fungus Sordaria macrospora
Source: PLoS One. 2009 Apr 13;4(4):e5177. doi: 10.1371/journal.pone.0005177 (PMC2664464; doi:10.1371/journal.pone.0005177)
Supplement: Table S1 — Oligonucleotides used in this study (0.08 MB DOC) [file pone.0005177.s003.doc]

**Table S1:** Oligonucleotides used in this study

|  | 5’🡪3’ Sequence |
| --- | --- |
| cynT1-a | TTGAGCTCCAACAATGCTTGG |
| cynT1-b | TATCAAAGAACACACCATGGAC |
| cynT1-c1 | CCGATCCAGACTGTTGTGACC |
| cynT1-d | ATGAACAACCCTACGATCCGG |
| cynT1-77 | GTCGGTGAGGATGGAAAATGTCAT |
| cynT1-79 | CTGTCGTCCACAAGAAGGTATGA |
| T3-1102 | CCTTACTGTTGCCGACCCTTAC |
| T3-1104 | GGCAAGATGGGCCTCAAGGGTG |
| cynT2-a | AGCAAGAAGGACATCACCATG |
| cynT2-b | GTCGCGGAAGTTGAAGACCCA |
| cynT2-c1 | GGGTCCTTGGCGACCTGCTCG |
| cynT2-d1 | GCCGCCCTCCAGCAGTCGTAT |
| cynT1-5f | GTAACGCCAGGGTTTTCCCAGTCACGACGATGGTGGGTTTCTCCCAAGTC |
| cynT1-5r | CAAAAAATGCTCCTTCAATATCAGTTAACATGAACAACCCTACGATCCGG |
| cynT1-3f | GAGTAGATGCCGACCGGGAACCAGTTAACCCGATCCAGACTGTTGTGACC |
| cynT1-3r | GCGGATAACAATTTCACACAGGAAACAGCCCAGAAGTCTGGTAGCGGTGT |
| cynT2-5f | GTAACGCCAGGGTTTTCCCAGTCACGACGCAGTTTGAAGCAACTGCATCG |
| cynT2-5r | CAAAAAATGCTCCTTCAATATCAGTTAACGCCGCCCTCCAGCAGTCGTAT |
| cynT2-3f | GAGTAGATGCCGACCGGGAACCAGTTAACGGGTCCTTGGCGACCTGCTCG |
| cynT2-3r | GCGGATAACAATTTCACACAGGAAACAGCGGCGAAGGAAGGTGATGCCGC |
| cynT3-5f | GTAACGCCAGGGTTTTCCCAGTCACGACGTGACGCCTGGACCGCGAGAAG |
| cynT3-5r | CAAAAAATGCTCCTTCAATATCAGTTAACGACGTTCAAGAATGAGGATGC |
| cynT3-3f | GAGTAGATGCCGACCGGGAACCAGTTAACGGTGTTGGTGTTGGTAGAGAG |
| cynT3-3r | GCGGATAACAATTTCACACAGGAAACAGCGATTTGCAAATGAATCCCACC |
| hph-f | GTTAACTGATATTGAAGGAGCATTTTTGG |
| hph-r | GTTAACTGGTTCCCGGTCGGCATCTACTC |
| ssu-f | ATCCAAGGAAGGCAGCAGGC |
| ssu-r | TGGAGCTGGAATTACCGCG |
| cynT1-RT_f | CTCTCAAGACGGTCCGCTAC |
| cynT1-RT_r | CGGATCGTAGGGTTGTTCAT |
| cynT2-RT_f | GAGCTCAACGTCTACGAGCA |
| cynT2-RT_r | TTCAGGTCCTTGAGCAGACC |
| cynT3-RT_f | GCTGGATTTCCTTCCTTTCC |
| cynT3-RT_r | AAACAACCCTCACCGTCTTG |
| cynT1-pQE-f | GGATCCTCTCCCGAGAACACCTTCCAC |
| cynT1-r | GTCGACCCATGGCCTGCGCAGCAGCAATCTCCGC |
| cynT2-pQE-f | GGATCCTTTAGCACACGTCTGGGTCTTTC |
| cynT2-r | AAGCTTCCATGGCGGCGGTCAGGTTATAGATCTTC |
| cynT3-pQE-f | GGATCCCCCGTCACCAACGAAGACATCG |
| cynT3-r | GTCGACCCATGGCAACAACCCTCACCGTCTTGCCCG |
| h3 | GTACTCGCCGATAGTGGAAAC |
| tC1 | CACCGCCTGGACGACTAAACC |
| cynT1-tC1 | GCTGCTTCGGTCGAAAGTCGTC |
| cynT1-h3 | GCAAAAGAAGATAGCATCCAG |
| T1-K | GACGTCACCGGGTTGCATGCCC |
| cynT2-tC1 | GCCAGTGAGATAAGTATAGGC |
| cynT2-h3 | GGGAGAAGAGGCGAAGAGGTG |
| T2-K | GAGCATCCGATCCAGAGATAC |
| cynT3-tC1 | GGTTCCAACGGAGGTGCTCCTCG |
| cynT3-h3 | CAAGGACTCGAGCAGCTTTGTGAAGG |
| T3-K | GGTGGAGGAGTAAGAAGCCG |
| cynT1-GFP-f | CCATGGTTTCTCCCGAGAACACCTTCCAC |
| cynT1-BamHI-r | GGATCCCTACTGCGCAGCAGCAATCTCCGCC |
| cynT2-GFP-f | CCATGGAATTTAGCACACGTCTGGGTCTTTC |
| cynT2-BamHI-r | GGATCCTTAGGCGGTCAGGTTATAGATC |
| cynT3-GFP-f | CCATGGAACCCGTCACCAACGAAGACATCG |
| cynT3-BamHI-r | GGATCCTCAAACAACCCTCACCGTCTTG |
| cynT2-CTG-f | GCGGCCGCCTGCGACTTAGACTCCCGAG |
| T1-Mut-f | GCGGCCTCGAGGTCCACGGTGTGTTCTTTGATATC |
| T1-Mut-r | GATATCAAAGAACACACCGTGGACCTCGAGGCCGC |
| CynT2_56f_NcoI | CCATGGAATCCCATAGCTCCCACTCCGAAAAAG |
| Mito-r | CCATGGCGTGGGAGCTATGGGAGCTGCAG |
| CTG-Mito_f | CTGCGACTTAGACTCCCGAGTGCTACCGC |
| pEHNnat-r | GGTGATGTCTGCTCAAGCGGGGTAGCTG |

Overhangs for homologous recombination in yeast are given in red. Nucleotides added as overhangs to introduce restriction sites are boxed in light blue and PCR based point mutations are underlined.
