# Supplementary material for: β-Carbonic Anhydrases Play a Role in Fruiting Body Development and Ascospore Germination in the Filamentous Fungus Sordaria macrospora
Source: PLoS One. 2009 Apr 13;4(4):e5177. doi: 10.1371/journal.pone.0005177 (PMC2664464; doi:10.1371/journal.pone.0005177)
Supplement: Table S2 — Plasmids used in this study (0.04 MB DOC) [file pone.0005177.s004.doc]

**Table S2:** Plasmids used in this study

| Plasmid | Characteristics | Reference |
| --- | --- | --- |
| pCAS1-GFP | *gpd*(*p*)::773 bp *Nco*I *cas2* restriction fragment::*trpC*(*t*) | This study |
| pCAS2-GFP | *gpd*(*p*)::858 bp *Nco*I *cas2* restriction fragment::*trpC*(*t*) | This study |
| pCAS3-GFP | *gpd*(*p*)::736 bp *Nco*I *cas2* restriction fragment::*trpC*(*t*) | This study |
| pΔMito-CAS2-DsRED | *gpd*(*p*)::699 bp *Nco*I *cas2* restriction fragment, deletion of 165 bp encoding for amino acids 2-55::*trpC*(*t*) | This study |
| pMito-DsRED | *gpd*(*p*)::183 bp *Nco*I *cas2* restriction fragment, encoding for amino acids 1-60 of CAS2::*trpC*(*t*) | This study |
| pDsRED-SKL | *gpd*(*p*)::*DsRed*::SKL::*trpC*(*t*) | [29] |
| pCAS1-KO | 1361 bp upstream and 860 bp downstream region for homologous recombination at the *cas1* locus separated by the *hph* resistance cassette | This study |
| pCAS2-KO | 762 bp upstream and 832 bp downstream region for homologous recombination at the *cas2* locus separated by the *hph* resistance cassette | This study |
| pCAS3-KO | 811 bp upstream and 970 bp downstream region for homologous recombination at the *cas3* locus separated by the *hph* resistance cassette | This study |
| pGPD-CAS1 | *gpd*(*p*)::775 bp *Nco*I/*Bam*HI *cas1* restriction fragment::*trpC*(*t*) | This study |
| pGPD-CAS2 | *gpd*(*p*)::860 bp *Nco*I/*Bam*HI *cas2* restriction fragment::*trpC*(*t*) | This study |
| pGPD-CAS2-CTG | *gpd*(*p*)::deletion of 105 bp encoding for the first 35 aa of CAS2::*trpC*(*t*) | This study |
| pGPD-CAS3 | *gpd*(*p*)::738 bp *Nco*I/*Bam*HI *cas3* restriction fragment::*trpC*(*t*) | This study |
| pCTG-Mito-DsRED | *gpd*(*p*)::deletion of 105 bp encoding for the first 35 aa of CAS2 mitochondrial target sequence::*trpC*(*t*) | This study |
| pEHN-nat1 | *nat1* expression cassette | U. Kück, unpublished data |
| pCYN1-KO | *hph* expression cassette | [58] |
| pRHN1 | *gpd*(*p*)::*DsRed*::*trpC*(*t*) | [73] |
